# Hyphal growth of *Penicillium rubens* in changing relative humidity

**DOI:** 10.1007/s00253-021-11343-6

**Published:** 2021-06-07

**Authors:** Philip Ruijten, Hendrik P. Huinink, Olaf C. G. Adan

**Affiliations:** 1 Department of Applied Physics, Eindhoven University of Technology, 5612 AZ Eindhoven, The Netherlands; 2 Netherlands Organization for Applied Scientific Research (TNO), High Tech Campus 25, 5656 AE Eindhoven, The Netherlands

**Keywords:** Dynamic relative humidity, Hyphal growth, Microscopy

## Abstract

**Abstract:**

When considering mold prevention strategies, the environmental conditions in which fungi grow need to be taken into consideration. This environment is often characterized by a time-dependent relative humidity, and porous substrate. Growth has mainly been investigated in steady-state experiments. Therefore, the goal of this study is to understand the hyphal growth of *Penicillium rubens* on porous gypsum, under dynamic humidity conditions. Spores of *P. rubens* were inoculated on porous gypsum containing nutrients, and placed in a small incubation chamber, allowing for microscopic hyphal observation. The relative humidity in this chamber varied multiple times between a high (close to 100%) and low value (35%, 55%, or 75%). The hyphae reacted to a lowered relative humidity by an immediate growth stop and dehydration. When the relative humidity was increased again, the hyphae re-hydrated and three responses were found: regrowing after approximately 4 h, after a time equal to the germination time, or no regrowth at all. No substantial regrowth was found for fluctuations faster than 4 h. This time-scale was found for multiple decreases in relative humidity, and has been reported for the first time.

**Key points:**

• *Hyphae restart growth after a characteristic time of approximately 4 h.*

• *Relative humidity fluctuations of 3 h can suppress hyphal growth.*

• *Hyphae do not regrow after a severe desiccation and short periods of high humidity.*

**Supplementary Information:**

The online version contains supplementary material available at 10.1007/s00253-021-11343-6.

## Introduction

Esthetic and medical problems can be associated with indoor mold growth (Flannigan 2001; Green et al. 2011; Miller 1992; Samson et al. 2010). In Europe, people spend approximately 90% of their time in indoor environments (Schweizer et al. 2006). According to the World Health Organization’s Large Analysis and. Review of European Housing and Health Status, WHO LARES, project, the indoor environment of 25% of all 3373 investigated dwellings (eight European cities: Vilnius, Geneva, Forli, Ferreira, Budapest, Batislava, Bonn, and Angers) suffer from mold growth in at least one room (WHO 2009). Prediction and prevention strategies for mold growth are thus needed.

The basis of such strategies lies in detailed knowledge of the colonization process of indoor surfaces. Many controllable factors such as moisture, nutrients, temperature, oxygen, and pH influence this colonization (Burgain et al. 2013; Grant et al. 1989; Griffin 1996; Sautour et al. 2001; van Laarhoven et al. 2015; Xie et al. 1997). A profound understanding of the effect on colonization of each of these factors is vital.

One factor which is essential for germination of all fungal spores is water (Adan et al. 2011; Gottlieb 1950). Studies on the relation between water and fungal growth are often studies done at constant humidity conditions. From these, it is known that a lower water availability results in an increased germination time and a decreased growth rate (Ayerst 1969; Burgain et al. 2013; Gervais et al. 1988; Judet et al. 2008; Nanguy et al. 2010; Segers et al. 2016). Indoor conditions encountered by fungi are characterized by porous substrates, e.g., concrete, wood, and gypsum (Andersen et al. 2011), as well as varying water-related conditions, e.g., due to temperature fluctuations, or household activities such as cooking or bathing (Adan et al. 2011). Therefore, there is a need for information on the fungal response to dynamic relative humidity conditions on porous media. The relative humidity, RH, is a measure of the water vapor in the air.

Several studies have focused on the dependence of macroscopic growth on a non-steady relative humidity environment on porous materials (Adan 1994; Bekker et al. 2015; Johansson et al. 2013; Segers et al. 2016; Viitanen and Bjurman 1995).

Complementary to the macroscopic studies executed, information on fungal cell level, i.e., microscopic information, can help to create prevention strategies. Microscopic studies in this type of environment are limited to a single decrease in relative humidity (Luard 1982b; Segers et al. 2016; van Laarhoven et al. 2016). It was concluded that hyphae can regain growth at a higher growth rate after a desiccation. Microscopic experiments with the more realistic situation of multiple wetting-drying environments are thus needed to fully understand hyphal growth after multiple desiccations.

The aim of this paper is to examine how hyphae of *Penicillium rubens* react to multiple periods of lowered relative humidity, while growing on a porous substrate. In this study, we vary the number of RH-fluctuations, the RH of the dry period, and the duration of the dry periods. An experimental set-up is used which allows for real-time microscopic observation of an incubation chamber, in which the RH can be varied between two defined values at well-defined moments in time. It is hypothesized that the hyphae will restart growth, depending on the characteristics of the desiccation. Results are also expected to give an indication of RH values needed in indoor environments that ensure no germination, making this environment unsusceptible to mold growth.

## Materials and method

### Fungal strain, spore harvesting, substrate, and inoculation

*P. rubens* was chosen as model organism, as it is a dominant indoor fungus, often colonizing indoor surfaces (Adan et al. 2011; Nevalainen et al. 2015; Verdier et al. 2014). It has a minimal water activity, *a*_*w*_, for growth of 0.82 at 25 ° C (Zalar et al. 2007; Segers 2017). In equilibrium, we have RH = a_w_ × 100%. Stock conidial suspensions of *P. rubens* (CBS 401.92; CBS Fungal Biodiversity Centre, Utrecht, the Netherlands, WDCM 133) with a concentration of 7 ∙ 10^6^ spores ml^−1^ were spread on autoclaved Malt Extract Agar (MEA). These MEA plates have a water activity, a_w_, of 0.995. These inoculated plates were incubated at 23 ° C in a climate room, until sporulation occurred, which consistently happened after 5 to 7 days. Conidia, developed on the MEA plates, were harvested with sterile cotton swabs (ClassiqSwabs, Copan Diagnostics, Murrieta, CA, USA).

The gypsum samples are inoculated with conidia from these plates using a cotton swab. Harvesting methods using solutions can have an influence on subsequent growth (Dantigny and Nanguy 2009; Nanguy et al. 2010; Nguyen Van Long et al. 2017; Nickerson et al. 1981). Therefore, dry harvesting is opted for.

The substrates for growth experiments were made by mixing gypsum (CaSO_4_∙1/2 H_2_O, Sigma Aldrich, Saint Louis, MO, USA) with an aqueous solution of Czapek Dox Broth (Oxoid, 8.76 gl^−1^, ThermoFisher, Waltham, MA, USA) and trace metals ZnSO_4_∙7 H_2_O (2.5 ∙ 10^−3^gl^−1^, Sigma Aldrich, Saint Louis, MO, USA) and CuSO_4_∙5 H_2_O (1.25 ∙ 10^−1^gl^−1^, Sigma Aldrich, Saint Louis, MO, USA). The solution was autoclaved, mixed with gypsum at a mass ratio of 2 : 3, and cast into 3 mm thick casts. These gypsum samples were dried for 48 h at room temperature in a Bio Safety Cabinet (BSC) (CleanAir, Class II–EF/B, Utrecht, The Netherlands) to remove excess water. The surface of each sample was colored by pipetting 5 μl of Fe_3_O_4_ suspension (33.3 gl^−1^ water, Metzger Black, Chempur, Karlsruhe, Germany), to provide sufficient contrast for the microscopy. This method is that of van Laarhoven et al. (2016).

### Set-up for growth experiments

**Fig. 1:**
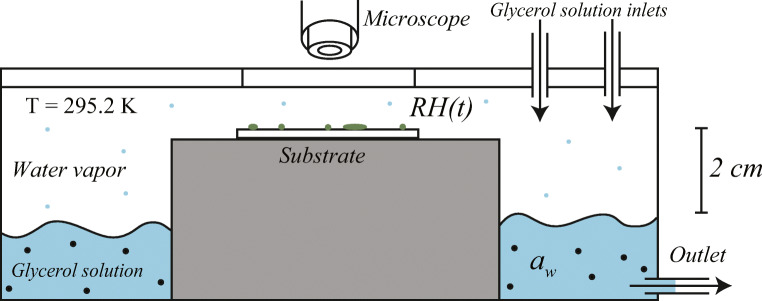
Schematic representation of the set-up for growth experiments. An inoculated sample is stored in the incubation chamber above a glycerol solution that controls the chamber RH. In equilibrium, we have RH = 100 %  ∙ a_w_, and the solution a_w_ followed from Forney and Brandl (1992). The RH in the container is controlled by the glycerol solution. Two inlets and one outlet allow for switching between two RH values. Growth on the substrate is recorded with video microscopy through the transparent cover of the chamber

Inoculated samples were placed in incubation chambers in our climate room, at 23 ° C, as seen in Fig. 1. Growth is observed through a transparent lid.

Tap water, creating an RH of just below 100%, was used during all “wet” periods, and thus referred to as RH_wet_. By using tap water, we ensure a consistent RH_wet_. It should be noted that at around 100% the growth rate is very dependent on RH: a slightly lower RH results in a relatively big change in growth rate (Segers 2017). To add to that, the RH is highly temperature dependent, so a small temperature fluctuation induces a significant fluctuation in RH, e.g., one degree difference in temperature corresponds to six percent RH difference. This choice of RH_wet_ thus entails some risks, but was measured periodically and our a_w_-meter (Labtouch-a_w_ Basic, Novasina AG, Lachen, Switserland), with an accuracy of 0.005, consistently read an RH of 100%. The temperature in our climate chamber was constant up to 1 ° C. The temperature in the inoculation chambers, however, varied less than 0.1 ° C, due to cooling of all electrical equipment present in the climate chamber. This was checked by thermocouple measurements (NI USB-9213, National Instruments, Austin, TX, USA). Regular checks for condensation were done, and no condensation was found in any experiment, ensuring a constant RH of just below 100%.

Following Forney and Brandl (1992), glycerol solutions were used to create the RH_dry_ values of the chambers during the experiments. To vary the relative humidity, the measurement chamber was emptied, flushed with the preceding water-glycerol mixture, and filled again to ensure that no residue was left behind. This was done using peristaltic pumps (BT100L, Lead Fluid). The resulting RH_dry_ was verified with a digital humidity sensor (SHT7X, Sensirion, Staefa, Switzerland). No residual solution was left between switching, and no splashing on the substrate was found.

During the dry periods, the RH was lowered to a lower value, indicated by RH_dry_. The mild RH value of 75% is below the minimal value of 86% for which growth can be sustained in the same climate room and analogous incubation chambers, on the same substrate (van Laarhoven et al. 2015). Modest and severe desiccations to 55% and 35% are imposed as well. These are the typical median and minimal values, respectively, of RH of the indoor environment, as found in literature (Telejko and Koruba 2019). These three values are therefore chosen as RH_dry_.

Analogous to van Laarhoven et al. (2015), growth was monitored by time-lapse recording of images. The time between two images was 1 h for all experiments considered. One hour is thus the experimental time resolution.

When switching between two liquids, i.e., switching between RH_dry_ and RH_wet_, two elements of equilibration have to be taken into consideration: the air, and the gypsum substrate. Adan (1994) showed that the surface moisture condition is critical for fungal growth, and focus will thus be on the surface of the growth substrate. In the case of an increase in RH to just below 100%, the surface a_w_ always reaches 0.95 within about 3 h, independently of the initial RH difference (van Laarhoven 2016).

Appendix A gives details about the equilibration of the vapor in the air surrounding the substrate and fungus. It is concluded that after a change in glycerol solution in the inoculation chamber, vapor equilibration is reached in a matter of seconds.

Two types of microscopes are used. A 7013MZT4 (Dino-Lite, Almere, The Netherlands) with magnification of 470×, corresponding to a field of view (FOV) of 0.84 mm × 0.63 mm, with pixels sized 0.6 μm × 0.6 μm, and an Edge AM 4515T8 (Dino-Lite, Almere, The Netherlands) with magnification of 900×, corresponding to a FOV of 0.4 mm × 0.3 mm, with pixels sizes 0.3 μm × 0.3 μm. Both have numeric aperture 0.22 and an optical resolution of approximately 1.5 μm. The focus depth of the microscopes is equal to 0.1 mm for the former and 0.07 mm for the latter.

### Experimental conditions and measured parameters

**Fig. 2:**
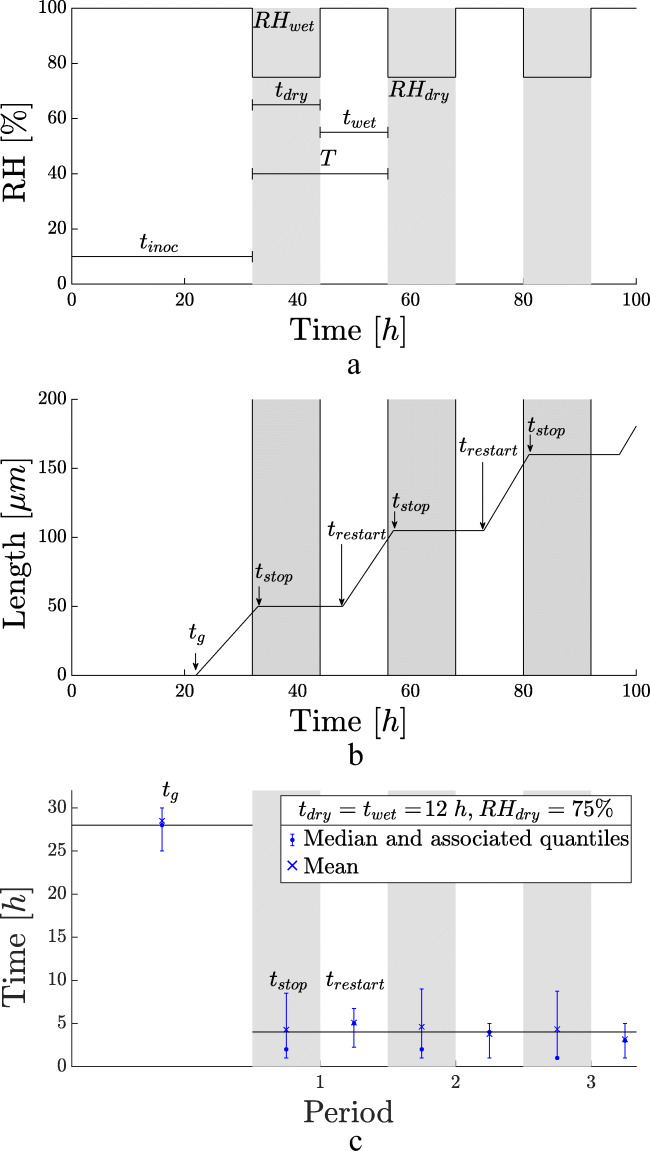
(**a**) Schematic representation of the dynamic RH during growth experiments. An inoculated sample is stored in the incubation chamber at high RH, i.e., RH_wet_, for a duration of t_inoc_ = 31 ± 1 h. After this germination period, the RH is lowered to RH_dry_ for t_dry_ hours. Subsequently, the RH is increased again to RH_wet_ for t_wet_ hours, and the cycle is repeated, resulting in a period T = t_dry_ + t_wet_. The gray areas show how dry periods will be indicated afterwards. (**b**) Generic hyphal growth curve, with germination, stop, and restart times, t_g_, t_stop_, and t_restart_ resp., and linear growth rate μ. The gray areas indicate the periods of RH_dry_. (**c**) Germination time t_g_, equal to 28 ± 2 *h*, stopping times t_stop_ (gray indicates RH_dry_), and starting times t_restart_ (white indicates RH_wet_) from the traced hyphae of Fig. 3: the first wet period is 32 h, and followed by periods of t_dry_=12 h (gray) and t_wet_=12 h (white). Note that t_g_ is indicated in the first period, while the other times are t_stop_ and t_restart_

In this section, the conditions of the conducted experiments are discussed, as well as the measured parameters. All experiments began at RH_wet_ for a time period of 31 ± 1 h, indicated by t_inoc_. This was done in order to ensure germination. Subsequently, a repetitive change in RH was applied, RH_dry_ for t_dry_ hours, and RH_wet_ for t_wet_ hours. Switching between RH_dry_ and RH_wet_ continued, with indicated duration t_dry_ and t_wet_ respectively, resulting in a period T ≡ t_dry_ + t_wet_. The time of wetness (TOW) is defined as TOW ≡ t_wet_/T, following the definition of Adan (1994). The parameters are depicted in Fig. 2a.

Table 1 shows the combination of RH_dry_, t_dry_, RH_wet_, and t_wet_ of all the experiments conducted. The results are divided by the three values chosen as RH_dry_.

**Table 1:** Parameters of experiments conducted, and associated growth rates

Experiment	RH_dry_ (%)	t_wet_ (h)	t_dry_ (h)	μ ± σ [μm/h]
Mild desiccation	75	14	10	9.5 ± 4
	75	8	8	6.9 ± 3
	75	12	12	10.8 ± 4
	75	8	16	11.5 ± 5
Modest desiccation	55	16	12	12.9 ± 7
	55	12	12	11.0 ± 4
	55	12	16	18.6 ± 8
Severe desiccation	35	24	32	15.5 ± 6
	35	12	12	8.2 ± 6
	35	12	16	8.8 ± 5
	35	8	16	14.8 ± 6
Variable fluctuation	75	3	3	No substantial growth
	75	12	12	13.2 ± 5
	75	3	3	No substantial growth

In each experiment, germination, restart, and stop times, t_g_, t_restart_, t_stop_, [h], resp., and linear hyphal growth rate, μ [μm/h], were monitored. These measured parameters are schematically represented in Fig. 2b. They are all determined from the hyphal length traced as a function of time.

The germination time, t_g_, is the time when no growth is visible for the last time. The time-parameter characterizing hyphal growth is the delay before growth restarts after switching from RH_dry_ to RH_wet_. This is indicated by the restart time, t_restart_. The time when hyphal growth stops during a period of RH_dry_ is obtained: the stopping time, t_stop_.

When the RH is high, i.e., RH_wet_, the hyphae grow and growth is measured and a linear fit is made for the growth curve, resulting in a linear growth rate. The linear growth rate was found to be 10.8 ± 4 μm/h.

The values of t_g_ and t_restart_ will be represented throughout this paper as in Fig. 2c. The dots represent the experimental median, the crosses represent the means, and the error bars are the associated first and third quartiles, as indicated in the figure legend.

The restarting times are shown in the white regions of RH_wet_. The horizontal black line at 4 h serves as a guide for the eye.

The stopping times t_stop_ are represented in the gray zones of RH_dry_. For all experiments considered, and for every period within an experiment, this stopping time was within the given experimental time resolution of 1 h, and will therefore not be shown.

Table 1 shows all the experiments conducted, with the associated growth rates.

### Statistical analysis

Each experiment was repeated at least five times. Approximately five to fifteen hyphae were traceable in one experiment. Post-processing of the captured movies with a custom MATLAB (The Mathworks ,Natick, Massachusetts, United States) script generated hyphal length, l, as a function of time, t, indicated by l(t). Any small change in camera position during the experiment, was also compensated with a custom MATLAB script. Growing hyphae could be followed until they grew out of the FOV, until the FOV was covered densely with hyphae thereby obscuring their tips, or until the experiment terminated. Following Dantigny et al. (2007), a normal distribution for these parameters was assumed. All results discussed consist of at least 25 hyphae, unless stated otherwise.

For the purpose of comparing the t_restart_ with t_g_, the function ttest2, MATLAB, with a significance level of 0.01 was used.

## Results

### Hyphal behavior

**Fig. 3:**
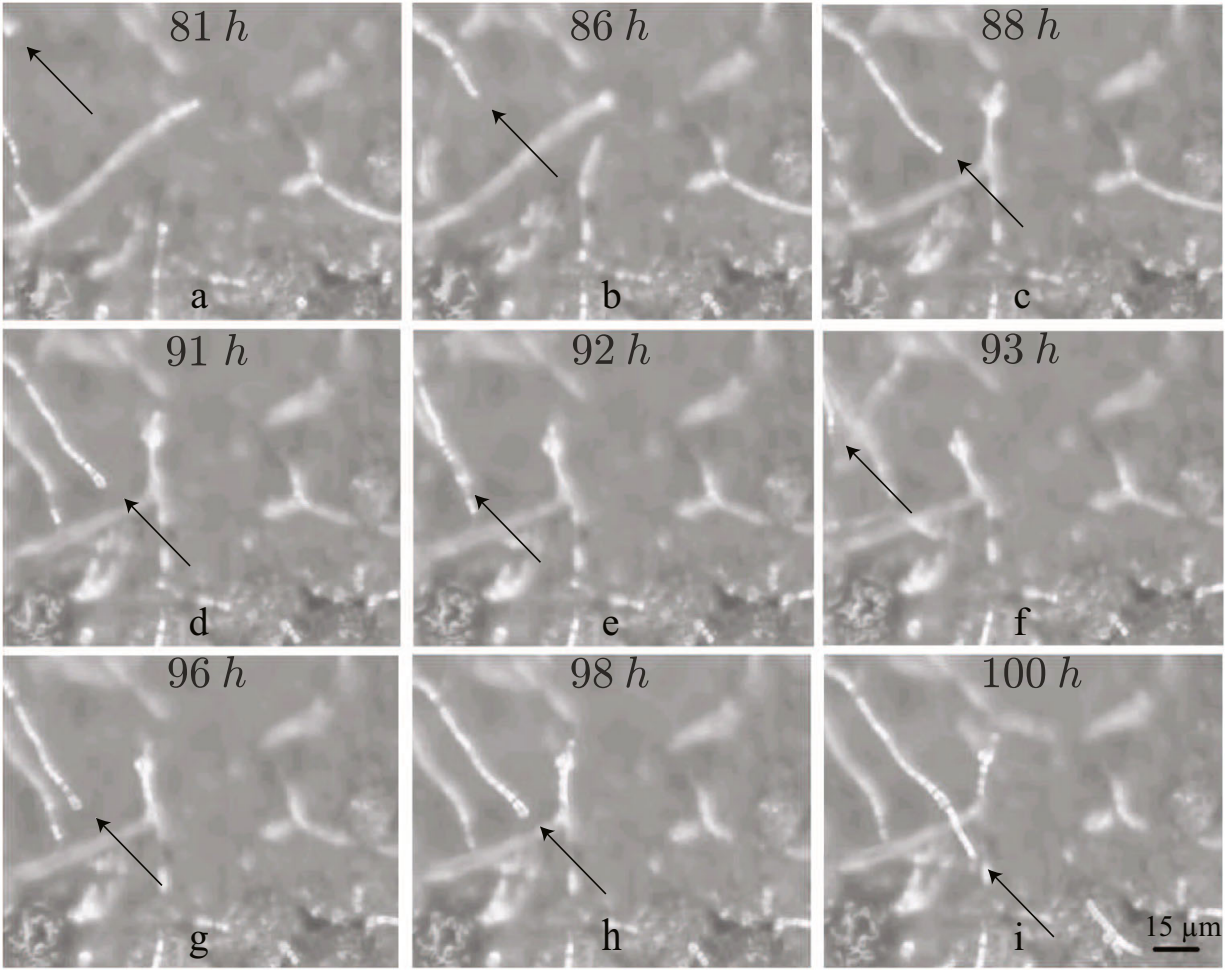
Images taken from the fourth and fifth cycle of the experiment with t_inoc_=32 h, t_dry_ = 6 h, t_wet_ = 8 h, and RH_dry_=75%. The first row, (**a**-**c**), taken at t=81 h, 86 h, and 88 h respectively, is characterized by RH_wet_. For the middle row, (**d**-**g**), are taken at t=91 h, 92 h, and 93 h, in an environment of RH_dry_. The last row, (g-i), are again taken in an environment with RH_wet_, taken at t=96 h, 98 h, and 100 h

To show how hyphae of *P. rubens* behave in the experimental set-up considered, a case study is presented. The experiment considered is characterized by: t_inoc_ = 32 h, t_dry_ = 6 h, t_wet_ = 8 h, and RH_dry_ = 75%. Figure 3 shows snapshots of the fourth and fifth period of the experiment. Supplementary material contains a video of the growth of hyphae on a gypsum substrate during this experiment. In a period of RH_wet_, Fig. 3a-c, a hypha grows. Figure 3d-f shows the hyphal response when the RH is changed to RH_dry_:the hypha stops growing within an hour. After this sudden stop, the hypha dehydrates and changes its position slightly due to shrinking.

When the RH is increased again, the hypha regains its former position, i.e., it rehydrates after which growth is restarted. Both rehydration and regrowth are seen in Fig. 3g-i. Regrowth starts after approximately 4 h.

This can be summarized as follows: after a decrease in RH, growth stops within 1 h. After this, dehydration takes place. Rehydration sets in once the RH is increased, and after approximately 4 h, growth restarts.

The growth rates found for each period, for all experiments, are found in Table 1.

### Periods of mild desiccation, RH_dry_ = 75%

Here, we discuss in more detail the impact of a repetitive change of RH, alternating between 100 and 75%. The changes in RH can be considered instantaneous, as mentioned in the “Materials and methods” section.

Four experiments are represented wherein t_dry_ has values of 14 h, 8 h, 12 h, and 8 h, with associated t_wet_ equal to 10 h, 8 h, 12 h, and 16 h, respectively.

**Fig. 4:**
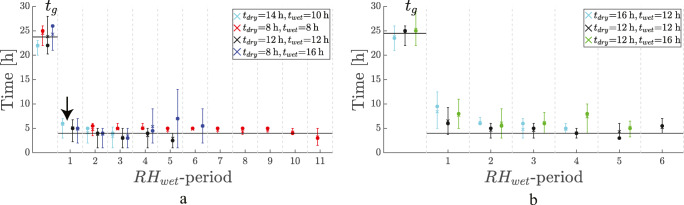
t_g_ and t_restart_ during experiments with RH_dry_ = 75% (**a**) and 55% (**b**). The horizontal axis indicates the RH_wet_ period, e.g., the first increase to RH_wet_ after RH_dry_ is indicated by “1,” etc. The values for t_dry_ and t_wet_ are shown in the legend. Dots indicate the median, crosses represent the mean, and error bars indicate the associated first and third quartiles. Note that t_g_ is indicated in the first period, while the other times are t_restart_. All data-points consist out of at least 25 hyphae. The horizontal line at 4 h is a guide for the eye, below which few hyphae started regrowing

In all the results shown in Fig. 4a, the number of hyphae was at least 25.

Four RH-cycles were carried out in the experiment with t_dry_ = 14 h and t_wet_ = 10 h. The experiment was terminated after this. For the other experiments, more cycles were carried out, until the field of view was so full of hyphae that their tips could not be traced anymore. Most switches between low and high RH could be monitored for the experiment with t_dry_ = t_wet_ = 8 h. This is because it took more cycles before the FOV was fully overgrown by hyphae.

For all four experiments, t_g_ falls between 20 and 28 h, and they are all similar. This was expected as the experimental conditions are identical. The average t_g_ is 24.5 h and indicated by the horizontal line.

During the subsequent periods of RH_wet_, the average start-times are similar, independent of t_wet_ and t_dry_, with the exception of the first wet period after a desiccation of 8 h, where no hyphae regrew. This is indicated by the arrow in Fig. 4a. In the first wet period after desiccation, no growing hyphae were registered. The horizontal line at 4 h is again a guide for the eye. In all experiments, very few hyphae started regrowing before this time.

The experiments with t_dry_ = t_wet_=8 h and t_dry_ = t_wet_=12 h have the same TOW, but a different period: T=16 h and T=24 h respectively.

The experiments with t_wet_=14, 12, and 8 h, and associated t_dry_=10, 12, and 16 h all have the same period T=24 h, but different TOW, namely, 0.42, 0.5, and 0.67 respectively. Although the TOW varied for these experiments, they show a similar t_restart_ at about 4 h.

The growth rates of all experiments are given in Table 1. They are all in the same order of magnitude: varying between 6.9 and 11.5 μm/h.

### Periods of modest desiccation, RH_dry_ = 55%

The results of a set of experiments with multiple decreases to RH_dry_ = 55% can be found in Fig. 4b. The t_dry_ has values of 16 h, 12 h, and 12 h, with associated t_wet_ equal to 12 h, 12 h, and 16 h, respectively.

In all the results shown in Fig. 4b, the number of hyphae was at least 25.

The experiment with t_wet_=16 h and t_dry_=12 h in Fig. 4b only shows four periods because the experiment was terminated after 150 h. The two other experiments, with more cycles, were stopped when the FOV was too cluttered with hyphae.

The shown t_restart_ values for all experiments with RH_dry_ = 55% are all similar and approximately 4 h. The horizontal line at 4 h is again a guide for the eye only: very few hyphae restart growing before this time-scale.

The growth rates of all experiments with RH decreases to 55% are given in Table 1. They are in the same order of magnitude: varying between 11.0 and 18.6 μm/h.

### Periods of severe desiccation, RH_dry_ = 35%

**Fig. 5:**
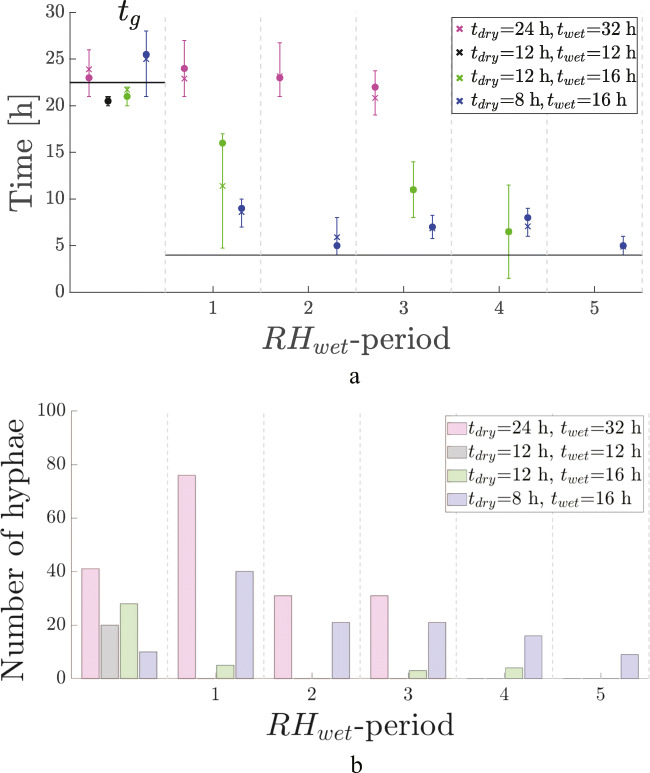
(**a**) t_g_ and t_restart_ during experiments with RH_dry_ = 35%. The horizontal axis indicates the RH_wet_ period, e.g., the first increase to RH_wet_ after RH_dry_ is indicated by “1,” etc. The values for t_dry_ and t_wet_ are shown in the legend. Dots indicate the median, crosses represent the mean, and error bars indicate the associated first and third quartiles. Note that t_g_ is indicated in the first period, while the other times are t_restart_. All data-points consist out of at least 25 hyphae. The horizontal line at 4 h is a guide for the eye, below which few hyphae started regrowing. (**b**) The corresponding number of traced hyphae per experiment and per period of the results shown in (**a**)

The growth behavior after multiple periods of RH_dry_ = 35% was investigated. The results are shown in Fig. 5a.

For a desiccation to 35%, three restart times are found: restart after a time-scale t_restart_ of about 4 to 10 h, regrowth with a longer time-scale of about 20 − 25 h, similar to t_g_, or no regrowth.

Firstly, the situation without regrowth is discussed: in the experiment with t_dry_ = t_wet_ = 12 h, no other hyphae grew outside of this first period of inoculation. For decreases to 75 and 55%, multiple periods of growth were found for these values of t_dry_ and t_wet_: no regrowth was seen within the wet periods of t_wet_ = 12 h, for a dry period of t_dry_ = 12 h. Although the experiment with t_dry_ = 12 h and t_wet_ = 16 h has data in multiple periods, the amount of hyphae there is considerably lower compared to the previous experiments. Only three-five hyphae were traced, as seen in Fig. 5b. This can be considered as very limited regrowth.

For t_dry_ is 8 h, with a t_wet_of 16 h, i.e., a short period of dehydration and a long period of rehydration and regrowth, significant growth is observed. This is shown in Fig. 5b. The time-scale of regrowth, t_restart_ is between 4 and 10 h, and the horizontal line at 4 h is again shown as a guide for the eye only: few hyphae restart growth below this time-scale.

The experiment with t_dry_ = 24 h and t_wet_ = 32 h shows significant growth. The t_restart_ for this experiment, however, is significantly larger: approximately 20 − 25 h, compared to the 4 to 10 h found for before. It is equal to the t_g_ found throughout.

The growth rates of all experiments with decreases to 35% are given in Table 1, they fall between 8.2 and 15.5 μm/h.

The number of hyphae traced in experiments with RH_dry_ equal to 75% and 55% involved at least 25. In this case, RH_dry_ is equal to 35% and number of hyphae traced is much less, as is shown in Fig. 5b.

### Changing RH fluctuations

Very little regrowth is seen for values of t_restart_ below 4 h. This is seen, by using the guide for the eye, in Fig. 3, 4, and 5. It is therefore investigated if growth is seen when t_wet_ = t_dry_ < 4 h.

**Fig. 6:**
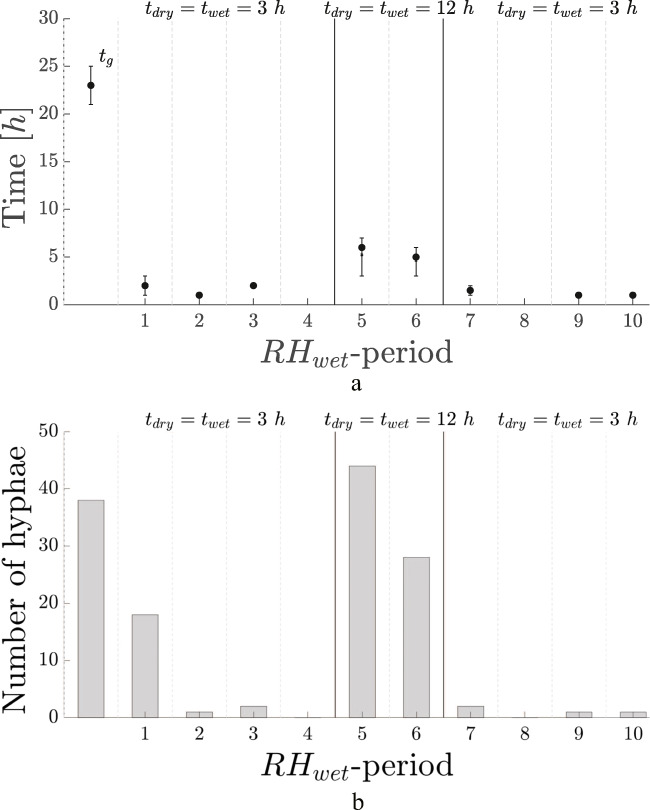
(**a**) Germination, and restart times of the experiment with RH_dry_ = 75%, and t_wet_ and t_dry_ as indicated. The horizontal axis indicates the RH_wet_ period, e.g., the first increase to RH_wet_ after RH_wet_ is indicated by “1,” etc. The dots indicate the median, crosses represent the mean, and error bars indicate the associated first and third quartiles. (**b**) The corresponding number of traced hyphae per experiment and per period of the results shown in (**a**)

An experiment was conducted with decreases to RH_dry_ = 75%, with varying t_dry_, and t_dry_ = t_wet_. First, four periods with t_wet_ = t_dry_ = 3 h were conducted, followed by two periods of t_wet_ = t_dry_ = 12 h, and again four periods of t_wet_ = t_dry_ = 3 h. The value for t_wet_ = 3 h is below the observed typical short time-scale of restart of 4 h. The restart times are shown in Fig. 6a.

In the first period after the inoculation, eighteen hyphae started growing within 3 h. In the fourth and eighth period, no regrowth is seen at all. In period 2, 3, 7, 9, and 10 very few hyphae regrew. Thus after period 1, no significant growth is seen in the periods with t_wet_ = t_dry_ = 3 h. This can be seen in Fig. 6b.

In the two periods with t_wet_ = t_dry_ = 12 h, regrowth is seen with a t_restart_ similar to all the t_restart_ values found before. The amount of traceable hyphae is again substantial, and the growth rate, found in Table 1, is around 13 μm/h, similar to values discussed in the previous sections.

After the two 12 h periods, four periods with t_wet_ = t_dry_ = 3 h followed. Again no significant growth is found.

## Discussion

Three different hyphal restart methods after a RH-decrease are found in the experiments conducted: regrowth after approximately 4 h, 25 h, or no regrowth at all. The short time-scale of hyphal regrowth is seen throughout all the sections in the “Results” section. This time-scale, approximately 4 h, is consistently found, for multiple values of of RH_dry_, t_wet_, t_dry_, T, and TOW. When the relative humidity is decreased, the hyphae lose water and turgor. When the relative humidity is increased again, the hyphae rehydrate. After this, regrowth is initiated. After a long and severe desiccation, i.e., big t_dry_ and low RH_dry_, regrowth is found after a t_restart_ equal to the germination time, or no regrowth is found within the investigated t_wet_.

Next to the restart time, the growth rates of all experiments were monitored. The growth rates were represented in Table 1, and are all in the same order of magnitude and similar to literature (van Laarhoven et al. 2016). A single decrease in RH of 1 h to 50%, 60%, 80%, 80%, and 90% resulted in increased growth rates, with a 2 μm/h increase from 8 μm/h. The porous substrate of the growth experiments was the same. The onset of the decrease in RH, however, was different to the onset of the first RH_dry_ in our work: 96 h by (van Laarhoven et al. 2016), vs. onset after 32 h in this work. Results presented here all have a first decrease in RH after 32 h. It was found by Bekker (2014) that the timing of the drought period had no influence on its effect, on a macroscopic scale: irrespective of the developmental stage of *P. rubens* prior to a RH decrease, the time after desiccation to sporulation remained the same.

Regrowth after a time-scale of approximately 25 h is only seen in the experiment with RH_dry_ = 35%, t_dry_ = 24 h, and t_wet_ = 32 h. This time-scale is significantly larger than the characteristic regrowth time-scale of 4 h and equals the germination time t_g_ found in all experiments. It is hypothesized that growth in this experiment is a consequence of new germination: a considerable decrease in RH, to RH_dry_ = 35%, continued for an extended time, t_dry_ = 24 h, prevents regrowth of hyphae. When this is followed by a lengthy period, t_wet_ = 32 h, of RH_wet_, ungerminated spores can germinate. This would then imply that there is a typical time period and dry RH value, after which hyphae cannot regrow. Hyphal autolysis might occur (Perez-Leblic et al. 1982), but more research is needed to confirm this.

Literature can be found on the response of fungi to changing humidity conditions, e.g., (Adan 1994; Blomberg and Adler 1992; Park 1982; Segers et al. 2016; Viitanen and Bjurman 1995; Viitanen and Ojanen 2007), but comparison between these studies is difficult due to temperature and substrate variation etc. (Dedesko and Siegel 2015; Vereecken and Roels 2012). The work of van Laarhoven et al. (2016) and Bekker (2014), however, can be compared with our results. The substrates used are the same, as well as the type of fungus.

**Fig. 7:**
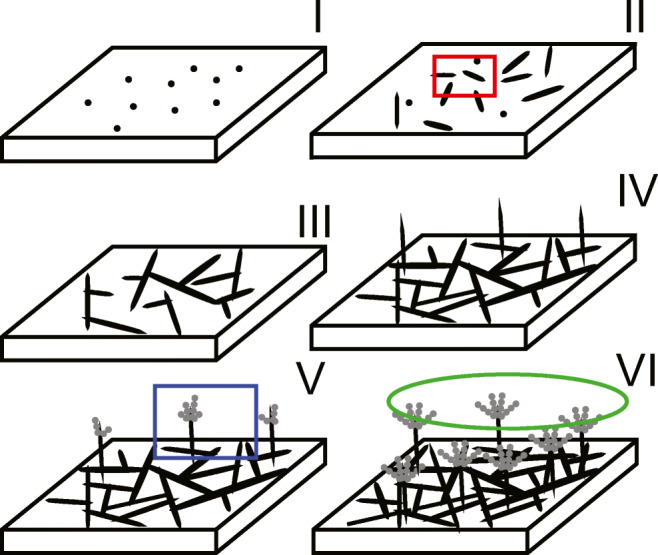
Overview of different development stages of *P. rubens* on gypsum, from (Bekker 2014): (I) ungerminated conidia (12 h), (II) onset and minimal germination (18 h), (III) germ tubes and branching hyphae (24 h), (IV) aerial hyphae (48 h), (V) initial conidiophore formation (72 h), and (VI) regular conidiophore formation (96 h). The highlighted regions are the scales of interest of the work by Bekker (2014) (green), (van Laarhoven et al. 2016) (blue), and the work represented here (red)

Bekker (2014) identified six different development stages for *P. rubens*. These stages are as follows: ungerminated conidia, onset and minimal germination, germ tubes and branching hyphae, aerial hyphae, initial conidiophore formation, and regular conidiophore formation. They are presented in Fig. 7. Bekker looked at the macroscopic scale by the discoloration by the formed conidia: two decreases in RH were given at different stages of development, resulting in a different discoloration. The results of Bekker can be summarized as follows: the timing of the decrease in RH, i.e., the stage of the development, affects its impact, and a 48 h-period of lowered RH, prior to sporulation, fully resets the development of *P. rubens*, while other structures can resume growth after a shorter desiccation. We hypothesize that a decrease of 24 h to 35% results only in new germination and not in regrowth of existing hyphae. Although our parameters are different, i.e., t_dry_=24 h (vs. 48 h, Bekker (2014)) and RH_dry_=35% (vs. 75%, Bekker (2014)), the concept is the same: a certain depth and length of desiccation stops regrowth of hyphae, and only new germination is possible. The work by Bekker (2014) indicates that other elements than spores regrow after a desiccation, which is confirmed by our results.

**Fig. 8:**
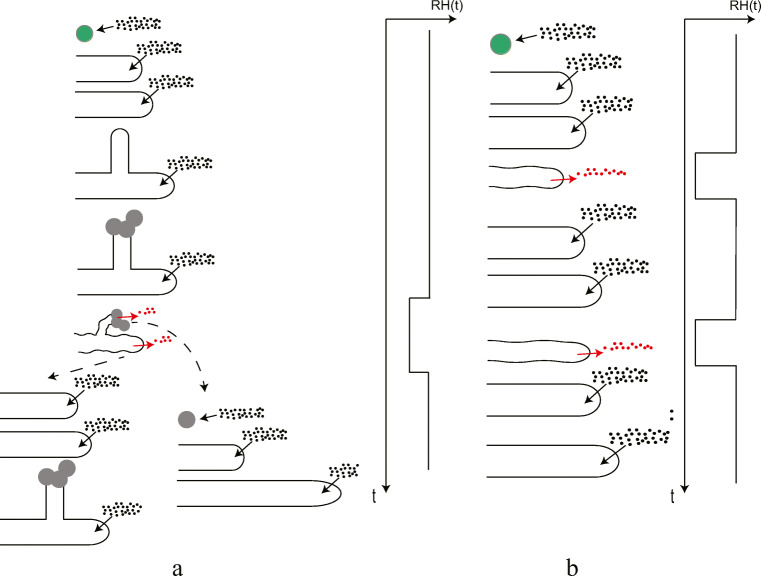
(**a**) Schematic representation of the experiments conducted by van Laarhoven et al. (2016), where a single decrease in RH was given after 96 h, when aerial hyphae and the first spores were already developed. The black dots indicate the water vapor, defining the RH. The red dots represent water expelled by the hyphae during dehydration. The new spores differ from the original spores present, and result in hyphae with a different growth rate. (**b**) Schematic representation of the regrowth response of a hypha. Multiple decreases in RH are applied to spores formed under one specific condition. The experimental parameters are RH_dry_, t_dry_, and t_wet_, as discussed. At the onset of a decrease in RH, the hypha loses water, shrinks, and stops growing within the hour. When the RH is increased again, regrowth starts

One of the few publications where hyphal level response was investigated in a dynamic environment is that of van Laarhoven et al. (2016). They investigated the mycelium at a scale indicated by the blue square in Fig. 7. As shown in the previous section, the method of regrowth depends on the depth, duration, and moment of application of the RH decrease. This is represented by the two options of regrowth in Fig. 8a. Regrowth of hyphae was found by van Laarhoven et al. (2016), but with a higher growth rate after the period of desiccation, compared to before, a clear difference from the results in this work. The decrease in RH imposed by van Laarhoven et al. (2016), with t_dry_ = 24 h, was initiated after 96 h, which corresponds to a decrease in the fifth growth phase, were sporulation starts. It has been shown that the spore formation conditions determine their water adsorption behavior (van Laarhoven et al. 2017) and physiological state (Nguyen Van Long et al. 2017). Further, it has recently been found that sporulation in stressed conditions affects growth (Ruijten et al. 2020). We therefore hypothesize that these faster growing hyphae observed by van Laarhoven et al. (2016) are the result of a “second generation spores,” as is depicted in Fig. 8b. Second generation spores are then non-inoculated spores, but grown in the experimental set-up. It was concluded by van Laarhoven et al. (2016) that “growing tips exposed to a desiccation of any considered duration or RH become unviable for further growth afterwards.” This conclusion is thus proven incorrect.

The result that hyphae can restart growth with a short time-scale of approximately 4 h will now be discussed. In our study, the dry periods are initiated when the culture is in the growth phase II from Fig. 7. Regrowth after this short time-scale follows the following behavior, shown in Fig. 3: hyphae germinate, grow, dehydrate and shrink down, stay motionless, rehydrate, and regrow with a characteristic short time-scale. This is represented in Fig. 8b. This time-scale can be attributed to the time needed for a hypha to refill itself and restart growth. The fact that this time-scale was observed in most experiments might be explained by a hyphal “dormant” state, reached below a certain threshold of RH. When the RH increases again, the time to go from this dormant state to a state of regrowth is approximately 4 h. Fungal spore dormancy is well-known (Griffin 1996), and more research needs to be done to explore this possible hyphal dormancy. Regrowth is a combination of both active and passive events with their respective time-scales (Steinberg 2007). An example of the former is molecular motors supplying cargo to the hyphal tip. Osmosis is an example of a passive process. The latter is influenced by the amount of internal osmolites in the hyphae, which is species-dependent (Luard 1982a). Experiments with other species should be conducted to test if the observed time-scale depends on this internal osmolyte composition. It has recently been found that microbial growth in carpet dust can be sustained in the indoor environment even with short 6-h bursts of elevated relative humidity in a room (Haines et al. 2020). This time of high RH is longer than the minimum needed to sustain fungal growth found in this work.

The growth rates from Table 1 can be explained by assuming that the typical growth rate of the hyphae is determined by the conditions during sporogenesis. Spores formed at low water activity results in more osmolites (Luard 1982a; van Laarhoven et al. 2017), which leads to the ability of growth in more stressing environments (Ruijten et al. 2020). The growth rate in indoor environments differ from the incubation chamber: dust can be present, paper surrounds gypsum, etc. The results presented here should thus not be compared to work where other factors might influence the growth rate. It is not known if these other factors also influence the time-scale of 4 h.

It is noted that the growth rate depends on the moisture content, which was not discussed in this work. It has been found that on clean surfaces, only the RH is enough for early hyphal growth (Ruijten et al. 2020). From the appendix, it followed that the surface RH reached fast equilibrium, triggering hyphal regrowth.

In conclusion, this study investigates the effect of relative humidity, RH, fluctuations on the hyphal growth of *P. rubens* on a porous substrate. The hyphal response to a decrease in RH at a microscopic level was observed. It was found to be a growth-stop within an hour: the hypha loses water and shrinks, before becoming static. When the RH is increased again, the result of all fourteen represented experiments can be categorized in three different responses, depending on RH_dry_, t_dry_, and t_wet_: regrowth after approximately 4 h, 25 h, or no regrowth at all.

Characteristic hyphal regrowth with a typical short time-scale, of about 4 h, has been observed for the first time. This regrowth with this short time-scale is found consistently in different experiments, and therefore considered characteristic for the fungal response to RH fluctuations. No regrowth below this characteristic time-scale is found.

It is hypothesized that the regrowth after approximately 24 h is a consequence of germination of ungerminated spores, and not hyphal regrowth. It is, however, stressed that this study does not prove that germination is the only source of growth after a long severe desiccation.

The final response is no regrowth, which is due to a long and severe desiccation, followed by a period of high RH, which lasts shorter than the germination time.

Hyphal regrowth at a microscopic level, after multiple decreases in RH, has been investigated for the first time, and refuted earlier findings. The importance of experiments at the microscopic scale mimicking a realistic environment thus lies in its use to unveil all hyphal response types to desiccation. The practical application of the results found here is linked to indoor RH recommendations: the indoor RH should be such that the period of growth-susceptible-RH does not last more then approximately 3 h. Other factors determining this general growth-susceptible-RH should be looked into. A pragmatic use of the results of this work also lies in fungal modeling: when considering the time-scales at which fungi react to an increased RH, any model should incorporate this time-scale of regrowth, which is the main finding reported.

Besides multiple fluctuations between two set RH values, other combinations of RH decrease, their duration, and that of a subsequent high RH period, could be included in future experiments. It has been found that the indoor fungus *Cladosporium halotolerans* survives humidity dynamics markedly better than *Aspergillus niger* and even *P. rubens* (Segers et al. 2016). Therefore, similar experiments with other species can give new information. Besides other transient RH conditions and species, novel compounds or materials that might inhibit growth of molds on indoor surfaces under simulated real conditions should be investigated.

### Supplementary information

ESM 1 (MP4 963 kb)

## Figures and Tables

**Fig. 9:**
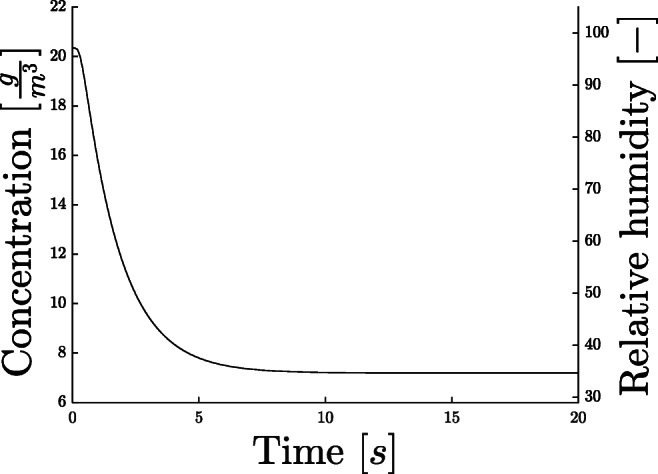
Evaluation of the first 1000 terms of Equation 1, for a switch from RH = 95 to 35%. Within a matter of seconds, the air in the experimental set-up of Fig. 1 is equilibrated after a change in RH

## Data Availability

Raw data were generated at Eindhoven University of Technology. Derived data supporting the findings of this study are available from the corresponding author H.P.H. on request.

## Appendix: air equilibration in the incubation chamber

For air equilibration, a diffusion constant of D = 0.28 cm^2^s^−1^ for water vapor in air is found in the literature (Cussler 2009). A RH step from 99 to 35%, at 23 ° C, corresponds to concentrations of c_i_ = 20.3 gm^−3^ and c_f_ = 7.2 gm^−3^ respectively. The general solution to the diffusion equation in this set-up, with d = 2 cm as indicated in Fig. 1, is given by:

1 $$ c\left(x,t\right)={c}_f+\frac{4}{\pi}\left({c}_i-{c}_f\right){\sum}_{n=0}^{\infty}\frac{{\left(-1\right)}^n}{2n+1}\mathit{\cos}\left(\frac{\left(2n+1\right)\pi x}{2d}\right){e}^{-\frac{\left(2n+1\right){\pi}^2}{2d} Dt} $$

Equation 1 has been evaluated, with 1000 terms, and the concentrations are shown in Fig. 9. In a matter of seconds, equilibration to the new RH has occurred. This fast air equilibration was also confirmed by van Laarhoven (2016).
